# Smartphone behavior and loneliness among older couples in China: Actor-partner interdependence modeling

**DOI:** 10.1371/journal.pone.0359013

**Published:** 2026-10-14

**Authors:** Siqi Huang, Wanjia Zhang, Yucen Xie, Bo Gao, Huan Zhou

**Affiliations:** West China School of Public Health and West China Fourth Hospital, Sichuan University, Sichuan, China; Prague University of Economics and Business: Vysoka Skola Ekonomicka v Praze, CZECHIA

## Abstract

**Background:**

Much evidence suggests that smartphone behavior is associated with loneliness, but no studies have examined the relationship at the dyadic level in Chinese older couples. The aim of this study is to determine whether smartphone behavior is associated with loneliness in individuals and their spouse.

**Methods:**

This cross-sectional study included 202 older couples from two community healthcare centers in Chengdu, China, between October 2022 and May 2023. The actor-partner interdependence model (APIM) was constructed to investigate the interdependent effect between smartphone behavior (i.e., smartphone proficiency, smartphone duration, smartphone usage, and overall smartphone behavior score) and loneliness.

**Results:**

In terms of actor effects, husbands’ smartphone proficiency, duration, usage, and overall behavior score were negatively associated with their own loneliness. For wives, however, only their smartphone proficiency, duration, and overall score showed such negative associations; no significant actor effect was found for wives’ smartphone usage on their own loneliness. Regarding partner effects, some significant findings emerged: husbands’ longer smartphone duration and higher overall behavior score were significantly associated with lower loneliness in their wives.

**Conclusions:**

This research indicated the dyadic interdependence of smartphone behavior and loneliness in older couples. Future mental health interventions for the older people should treat the couple as a unit.

## Introduction

Loneliness, which is defined as an unpleasant subjective feeling of social isolation or lack of companionship [1], has been a major public health concern for people of all ages [2,3], particularly older people who are more vulnerable to it and experience worse health outcomes, including lower well-being, chronic diseases, cardiovascular disease, depression, dementia and even death [4].

Loneliness is related to many factors, such as female gender, older age, low income, marital status and social interaction, among others [4]. In terms of social interaction, the smartphone has become an indispensable tool for almost everyone in China. Not only does it have various instrumental functions (e.g., making phone calls, sending text messages, chatting on WeChat or QQ, taking photos and paying bills), but it also has wonderful entertainment functions (e.g., listening to music, watching videos, watching the news and playing games), as well as learning functions that allow people to conveniently access a variety of educational information [5].

Studies have shown that loneliness can be associated with smartphone use, but this effect is paradoxical and inconsistent. On the one hand, Rotondi et al. found that the increase in smartphone use may be negatively associated with the quality of face-to-face social interactions and correlated with increased loneliness, arising some concerns about smartphone use [5]. On the other hand, Petersen et al. reported that increased daily smartphone use was associated with reduced loneliness by facilitating social interaction (despite not face-to-face), particularly among socially isolated older people [6]. However, in a cross-sectional study of 1318 middle-aged Japanese adults, Wang et al. found a link between smartphone use and loneliness, depending on the way in which people used their smartphones, i.e., people who used their phones for social reasons reported less loneliness, while those who used their phones to pass the time felt more lonely [7]. Therefore, the relationship between smartphone use and loneliness needs to be further studied.

Although the older people are not the most active users of smartphones and their smartphone behavior is usually overlooked, both the number of older smartphone users and the amount of time they spend on smartphones per day are increasing rapidly with the popularity of smartphones [7]. This has led to calls for further research to better understand the relationship between smartphone behavior and loneliness in older people, for whom this may be a good way to protect against loneliness.

Previous research on loneliness and smartphone behavior in older people has focused on individuals [6–9], with very little research on older couples.As a special group, considering the special influence of emotional connections, interaction patterns, and shared experiences that older couples have formed over a long period of time living together on each other's psychology and behavior [10–12], the mechanism of their mutual influence in the process of smartphone use has not been fully explored. However, multiple studies have documented that couples’ emotions and behaviors can influence each other [13], for example, loneliness in one spouse can related to loneliness in the other [14,15], Ermer and Proulx also found that greater relationship strain was associated with spousal loneliness for both wives and husbands [16]. For Chinese older people, spouses have a greater impact on their loneliness than other people such as children, relatives, friends, etc [17].Therefore, the interdependence of couples should not be ignored [2], and more accurate statistical analysis is also needed, as traditional parametric statistics designed for independent individuals are inappropriate for non-independent individuals such as couples.

To investigate whether both of husbands’ and wives’ smartphone behavior is associated with their own and their spouse’s loneliness in Chinese older couples. This study uses the APIM model as a metatheoretical framework (Fig 1) [18], which is particularly suitable for analyzing how individual behaviors in a couple's dichotomous relationship affect psychological outcomes through the dual path of the actor effect and the partner effect. In the model, the significant actor effect represents the ability of a good husband or wife's own smart time use behaviors to influence their level of loneliness, and the partner effect represents the ability of a husband's or wife's smart phone use behaviors to influence each other's level of loneliness.This approach, which recognizes that individuals within couples are interdependent and their behaviors influence each other, can provide insights into the understanding of older adults couples’ smart phone use and loneliness interactions in terms of smartphone use and loneliness. Overall, based on the above findings, we hypothesize that higher levels of smartphone use by husbands and wives are associated with lower levels of loneliness for themselves and their spouses.

**Fig 1:**
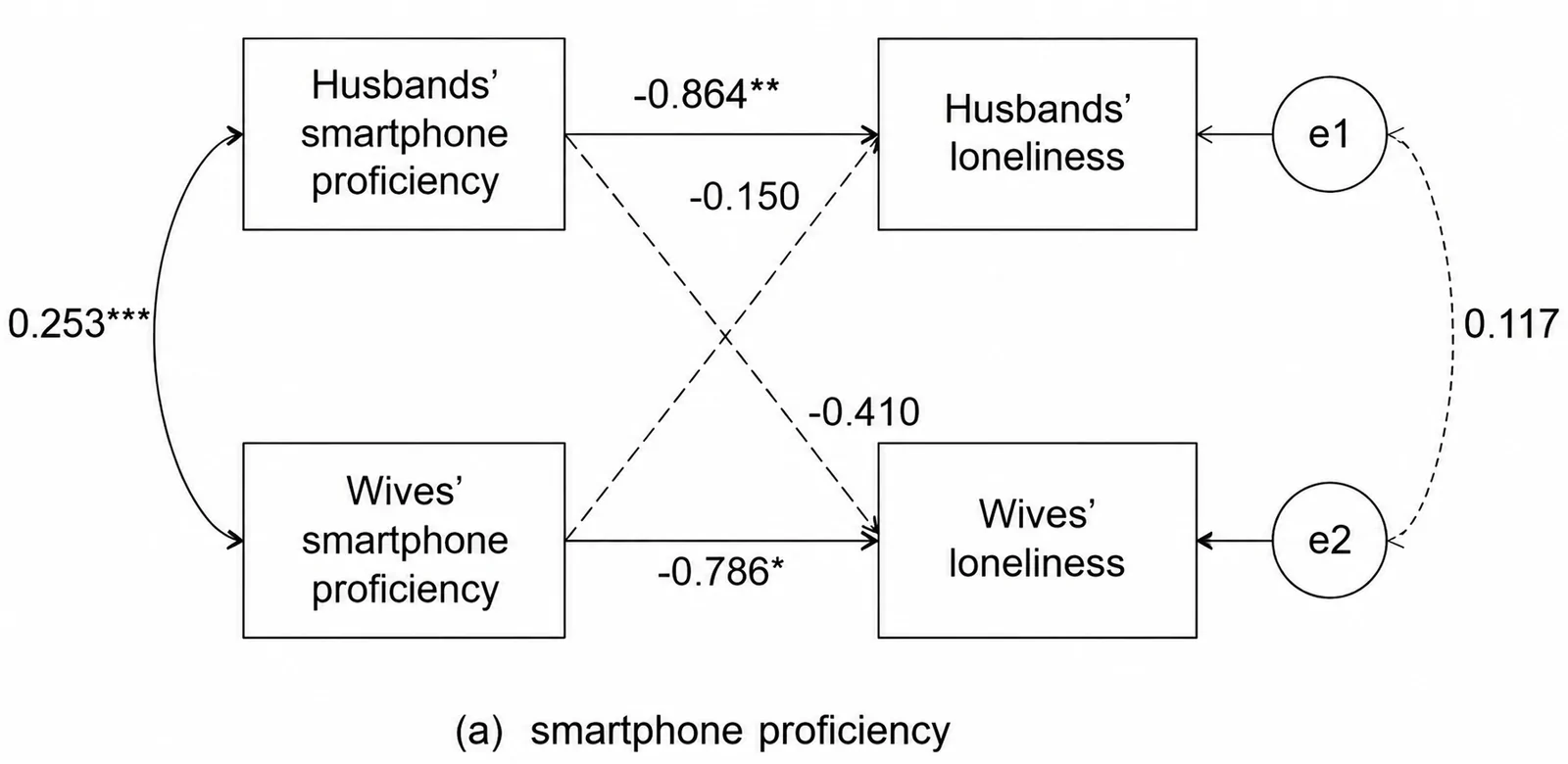
APIM plot of the relationship between loneliness and smartphone proficiency among older couples.

## Methods

### Study design

This cross- sectional survey was conducted in two community healthcare centers in Chengdu, China, between October 2022 and May 2023. The inclusion criteria for couples were: (1) age 60 years or older, (2) current smartphone users. After obtaining written informed consent, couples were interviewed separately face-to-face by trained interviewers and subsequently matched by their spouses’ names and phone numbers. For structural equation modeling with dyadic data, sample size adequacy depends on model complexity and the number of estimated parameters. In the present APIM, the number of free parameters is relatively small. Kenny, Kashy, and Cook [19] recommend at least 100–200 dyads for stable estimates in such dyadic models. Our sample of 202 couples exceeds this threshold. Although Kline [20] cautions against rigid minimum-N rules and generally advocates for larger samples in complex models, our N of 202 is considered sufficient for this simple path model (see also Wolf et al., 2013, for simulation-based recommendations) [21].211 couples were surveyed on-site, of which 7 couples refused to take the survey and 2 couples failed the quality of the questionnaire, resulting in a total of 202 couples included. Ethics approval was obtained from the Ethics Committee West China School of Public Health and West China Fourth Hospital, Sichuan University.

### Measures

The self-report questionnaires included questions on demographics, smartphone behavior and loneliness. Demographic information consisted of couples’ age, gender, and annual income(Full details are given in Appendix).

#### Dependent variable.

The couples’ current level of loneliness was measured using the 8-item University of California Los Angeles(ULS-8) Loneliness scale, which has been validated in Chinese older people [22], and included eight items:(1)I lack companionship,(2)There is no one I can turn to,(3) I am an outgoing person(reverse item),(4)I feel left out,(5)I fell isolation from others,(6) I can find companionship when I want it(reverse item),(7)I am unhappy being so withdrawn, and(8)People are around me but not with me. Each item was rated on a 4-point scale, where 1 = never and 4 = always. The scores were summed to give a loneliness score ranging from 8 to 32, with higher scores indicating greater loneliness. It’s the overall scores that were included in the final analysis, not the individual items, so loneliness was treated as a continuous observed variable rather than a latent variable.Cronbach’s α for this measure was 0.870.

#### Independent variables.

As the study population is older adults, the measurement of technology use needs to take into account ease of use and multidimensionality. The framework for measuring smartphone usage is therefore based on the UTAUT model [23]. Three complementary dimensions were selected for the self-designed questionnaire: proficiency (ability), duration (intensity) and software type (breadth). The following three questions were used to assess smartphone usage: (1) How well do you use the smartphone? i.e., smartphone proficiency, and the item was scored by responding “1 =a little, 2 =general, 3 =well, or 4 = very well”. (2) How much time do you spend on your smartphone per day? i.e., smartphone duration, response options for couples were 1(less than 1 hour), 2(1–2 hours), 3(3–4 hours), and 4(more than 5 hours). (3) What do you usually use your smartphone for? Couples were able to select more than one of the following options: “making phone calls”, “chatting on Wechat”, “chatting with QQ”, “reading news”, “watching videos”, “watching health lectures or information”, “playing games” etc. The smartphone usage score was calculated by counting the number of types selected from seven predefined categories (excluding an open ‘others’ option, which was not scored). Since all participants were current smartphone users and each selected at least one predefined type, the usage score ranged from 1 to 7. Thus, the overall smartphone behavior score (proficiency 1–4 + duration 1–4 + usage 1–7) ranged from 3 to 15, with higher scores indicating more extensive smartphone use. This composite score was constructed to provide a parsimonious summary of multidimensional smartphone behavior among older adults, consistent with the conceptualization of technology use as a multifaceted construct in the UTAUT framework [23].

### Statistical analysis

Comparison of demographics information, smartphone behavior and loneliness for husbands and wives were done using the paired t test or McNemar’s χ^2^ test. Normality and nonindependence of these variables were tested by kurtosis, skewness and Pearson’s correlation coefficient. To identify the actor and partner effects of the smartphone behavior on older adults couples’ loneliness, we constructed four APIMs using the structural equation modelling approach, with loneliness as the dependent variable, annual income as a control variable in four models, and smartphone proficiency, smartphone duration, smartphone usage, and overall smartphone score as independent variables for each model. The analysis was performed using SPSS 22.0 and AMOS 24.0.

## Results

### Descriptive of the couples

Table 1 showed the characteristics of the 202 pairs of couples. On average, the husbands were 2.32 years older than wives(*P* < 0.001). 90%of the older adults had an annual income of less than 100,000 yuan, and husbands tended to earn more than wives(*P* < 0.01). There were no significant differences in all domains of smartphone behavior and loneliness between husbands and wives(*P* > 0.05).

**Table 1:** Descriptive statistics of study variables (n = 202 couple dyads).

Variables	Husbands	Wives	t/χ^2^	P value
Age(years) M ± SD	73.55 ± 5.65	71.23 ± 5.06	9.056	<0.001***
Annual income (CNY)N (%)				
<50,000	97(48.02)	133(65.84)	19.262	0.004**
50,000-100,000	90(44.55)	57(28.22)		
100,000-150,000	9(4.46)	10(4.95)		
>150,000	6(2.97)	2(0.99)		
Smartphone proficiency N(%)				
A little	59(29.21)	51(25.25)	3.081	0.799
general	67(33.17)	75(37.13)		
well	50(24.75)	57(28.22)		
Very well	26(12.87)	19(9.41)		
Smartphone duration (hours)N(%)				
<1	30(14.85)	30(14.85)	2.422	0.877
1-2	74(36.63)	84(41.58)		
3-4	70(34.65)	60(29.70)		
≥5	28(13.86)	28(13.86)		
Smartphone usage M ± SD	2.74 ± 1.13	2.90 ± 1.22	−1.523	0.129
Overall smartphone score M ± SD	8.43 ± 2.41	8.54 ± 2.25	−0.542	0.588
Loneliness M ± SD	12.60 ± 4.28	12.88 ± 4.57	−0.697	0.486

Note. M±SD=mean ± standard deviation. *P<0.05, **P<0.01, ***P<0.001.

### Normality and correlation analysis

The correlations, kurtosis and skewness were depicted in Table 2. The correlation results showed that husband’s smartphone indicators were negatively associated with his and his wife’s loneliness (*r*s = −0.299 ~ −0.212, *P*s < 0.001), except for smartphone duration (*r* = −0.131, *P* > 0.05). The wife’s smartphone indicators were negatively correlated with her own loneliness (*r*s = −0.239 ~ −0.164, *P*s < 0.05), but not with her husband’s loneliness (*P*s > 0.05). What’s more, significant bivariate correlations were found for all smartphone indicators of couples (*P*s < 0.01), suggesting that the predictor variables were not independent, in which case APIM was more appropriate than traditional analysis [18]. As all absolute values of skewness and kurtosis were less than 2, the normal distribution assumption (i.e., the skewness was ≤ 3, and kurtosis was ≤ 10) was met [24], therefore, maximum likelihood estimation was used for the APIM analysis.

**Table 2:** Correlations among variables of husbands(H) and wives(W)(n = 202couples).

Variables	Skewness	Kurtosis	1	2	3	4	5	6	7	8	9	10
**1Loneliness(H)**	0.299	−0.030	1									
**2Proficiency(H)**	0.329	−0.991	−0.212**	1								
**3Duration(H)**	0.034	−0.782	−0.255***	0.394***	1							
**4Usage(H)**	0.438	0.378	−0.243***	0.368***	0.555***	1						
**5Overall score(H)**	0.144	−0.402	−0.299***	0.738***	0.802***	0.831***	1					
**6Loneliness(W)**	0.108	−0.236	0.155*	−0.131	−0.182*	−0.155*	−0.196**	1				
**7Proficiency(W)**	0.261	−0.830	−0.088	0.253***	0.159*	0.134	0.228**	−0.192**	1			
**8Duration(W)**	0.183	−0.734	−0.096	0.020	0.206**	0.171*	0.166*	−0.178*	0.225**	1		
**9Usage(W)**	0.873	1.133	−0.014	0.014	0.112	0.256***	0.168*	−0.164*	0.170*	0.519***	1	
**10Overall score(W)**	0.474	0.460	−0.082	0.120	0.209**	0.262***	0.252***	−0.239**	0.596***	0.776***	0.818***	1

Note. *P < 0.05, **P < 0.01, ***P < 0.001

### Actor and partner effects of smartphone behavior on loneliness

According to univariate correlation analyses, four separate APIM models were built:(a) smartphone proficiency on loneliness, (b) smartphone duration on loneliness, (c) smartphone usage on loneliness, (d) overall smartphone score on loneliness. Results of the final analytical model were presented in Table 3 and Figs 1, 2, 3, 4. Three APIM models suggested adequate overall fit (χ^2^/df:1–3, CFI > 0.9, RMSEA<0.08) [25].However, the duration model (Model b) did not meet the recommended fit thresholds (CFI = 0.829, RMSEA = 0.086), indicating poor model fit.The corresponding path diagrams for smartphone duration, smartphone usage, and overall smartphone score are shown in Figs 2–4, respectively.

**Table 3:** Non-standardized ESM coefficients of APIM.

Models	Actor Effects		Partner Effects		Goodness of fit		
lower	H→H	W→W	W→H	H→W	χ^2^/*df*	CFI	RMSEA
**Proficiency**	-0.864**	-0.786*	-0.150	-0.410	1.316	0.958	0.040
**Duration**	-1.143***	-0.750*	-0.191	-0.763*	2.481	0.829	0.086
**Usage**	-0.952***	-0.484	0.192	-0.461	1.201	0.975	0.032
**Overall score**	-0.522***	-0.413**	-0.006	-0.278*	1.852	0.903	0.065

Note. H: husband; W: wife; CFI: Comparative Normed of Fit Index; RMSEA: Root Mean Square Error of Approximation; *P < 0.05, **P < 0.01, ***P < 0.001.

**Fig 2:**
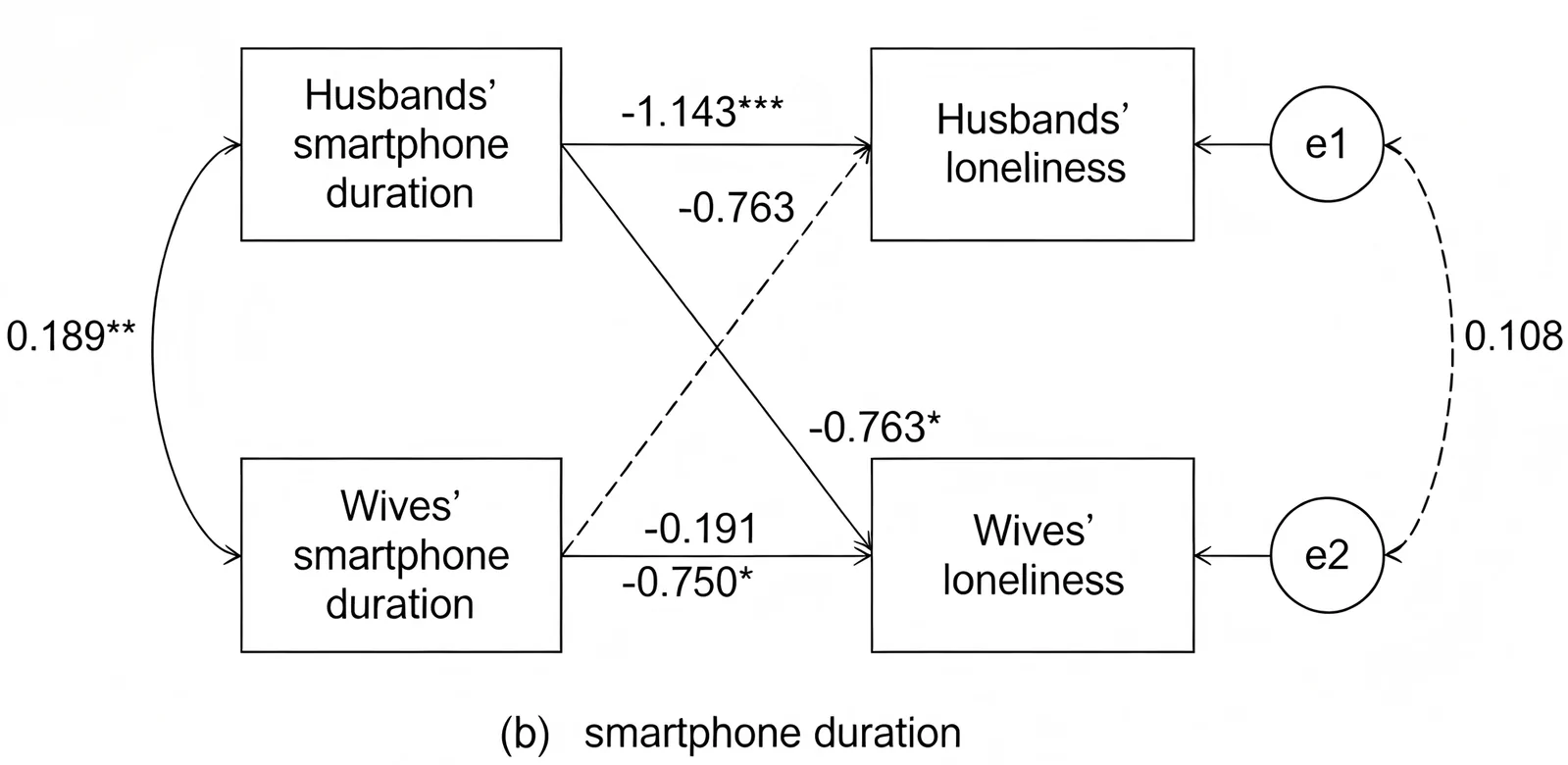
APIM plot of the relationship between loneliness and duration of smartphone use among older couples.

**Fig 3:**
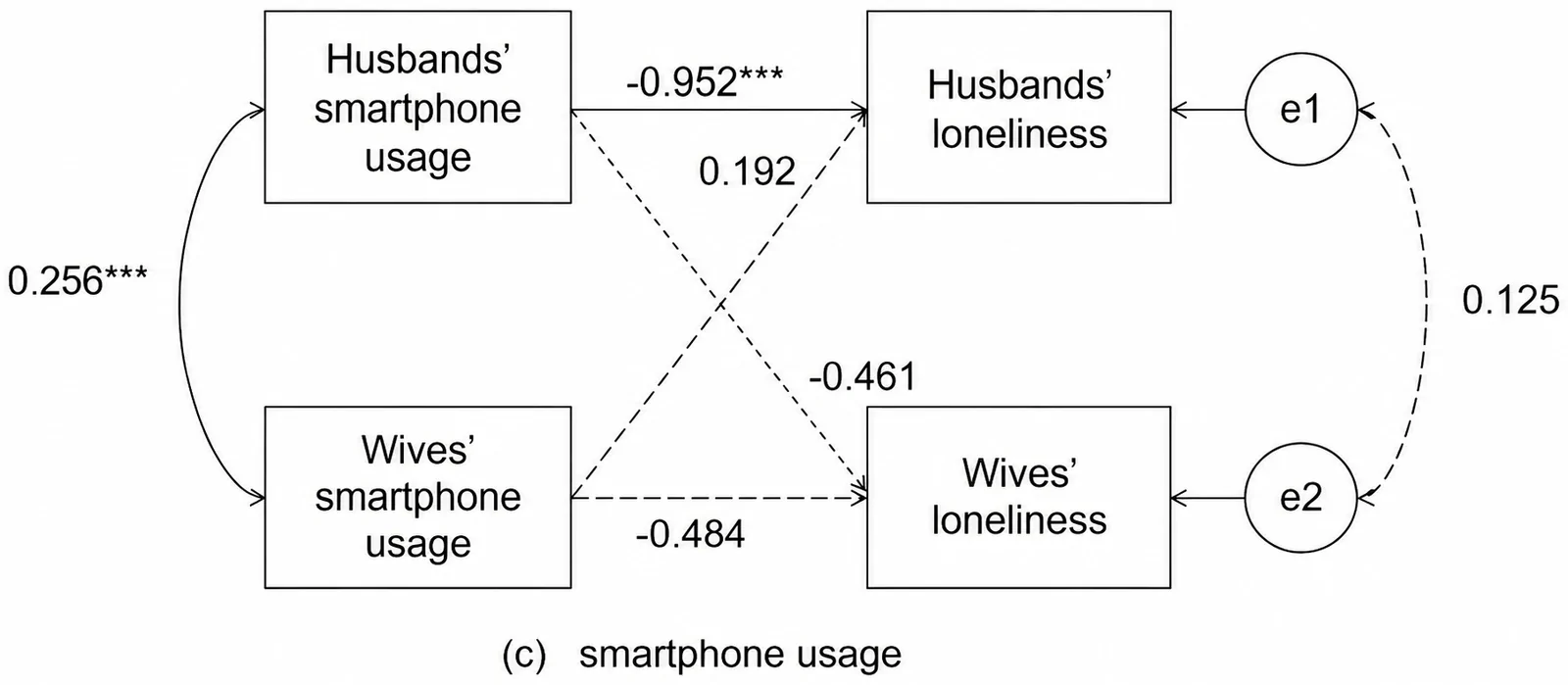
APIM plot of the relationship between loneliness and smartphone use among older couples.

**Fig 4:**
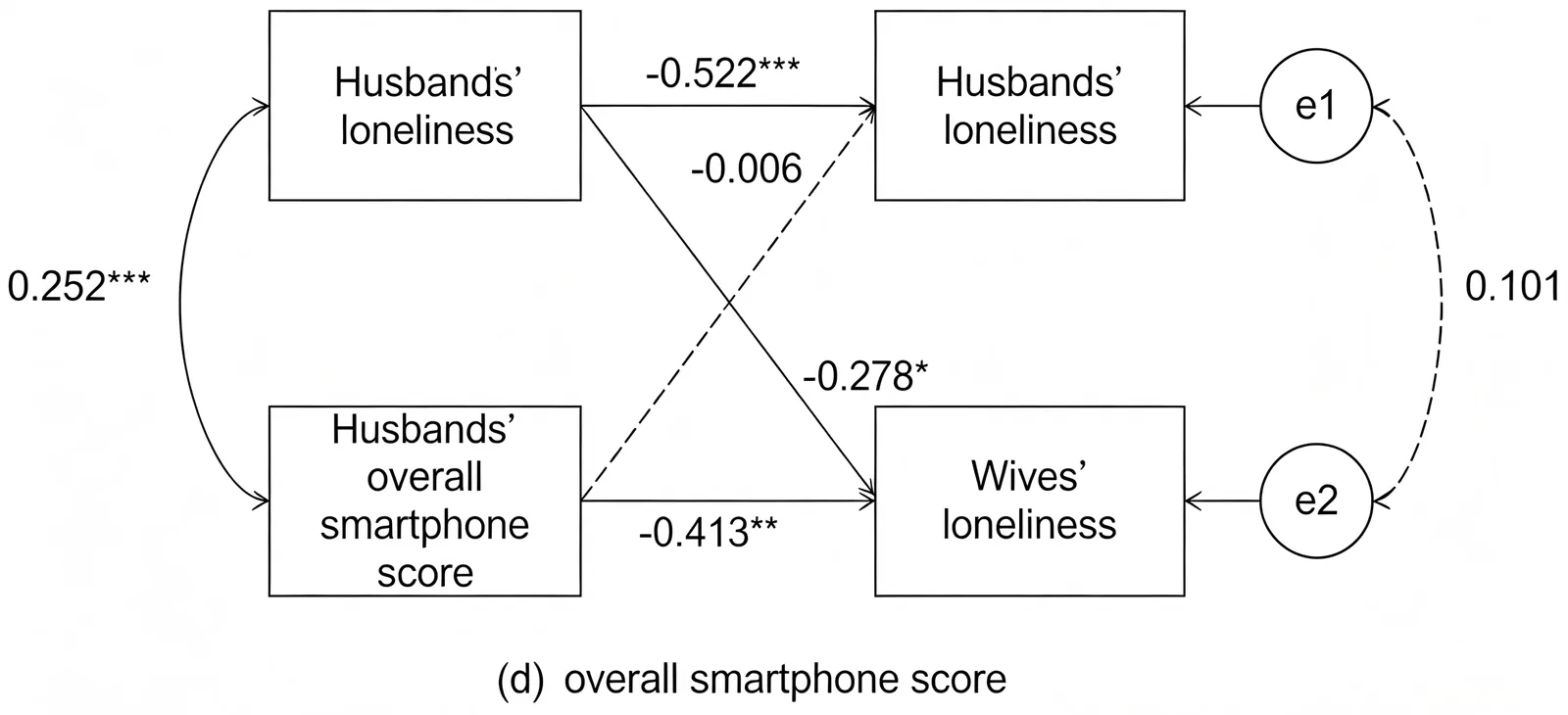
APIM plot of the relationship between loneliness and overall smartphone score among older couples. Notes: annual income was added as a covariate; non-standardized path coefficients were reported; significant effects were shown by the solid line, while non-significant effects were shown by the dashed line. *P < 0.05, **P < 0.01, ***P < 0.001.

### Actor effects

APIM actor effects indicated that almost all smartphone behavior indices for both husbands and wives were negatively associated with their own loneliness (*β*s = −1.143 ~ −0.522, *P*s < 0.01). However, for smartphone usage, only the significant actor effect of husbands’ smartphone usage on their own loneliness was observed (*β* = −0.952, *P* < 0.001).

### Partner effects

There were only two significant partner effect from smartphone behavior to loneliness. Specifically, longer husbands’ smartphone duration was associated with lower wives’ loneliness (*β* = −0.763, *P* < 0.05), and the spouses of husbands with higher overall smartphone score were more likely to have lower loneliness (*β* = −0.278, *P* < 0.05).

## Discussion

This study is the first to systematically reveal the effects of smartphone behavior on lonely actors and partners in older couples through the use of the APIM framework. Although an increasing amount of studies have investigated the relationship between smartphone and loneliness [6,7,9,26–28], yet these studies took the individual as the unit of analysis and failed to recognize that couples are not independent, as they usually have close interactions in the family system [29,30]. Most older couples would reduce their social networks, and focus time and attention on their closest relationships after retirement, as such, close relationships with their spouse may be particularly important for the overall health of both older husbands and wives [31,32], and in hence it is essential to consider couples as a unit of study. In this case, consider that own and spouse’s smartphone behavior may contribute independently to loneliness [18]. Compared to other research models, APIM is more suitable for capturing this close and complex interdependence between older partners, providing a more relevant and effective tool for studying older partner relationships. Thus, we used the appropriate APIM method to analyze couple dyads and found that actor effects existed for both husbands and wives, but partner effects existed only for wives’ loneliness.

According to the interdependence theory, interdependence increases with long-term commitment [33]. Subsequently, some empirical studies have also found that couples become more and more similar in attitudes and emotions over time [33–36]. Similar with these studies, our study showed that there were no significant differences(P > 0.05) but modestly correlations (rs < 0.50)and interdependencies in smartphone behavior and loneliness between husbands and wives, indicating that the older couples tended to have similar behaviors as well as feelings under the influence of each other.

As for actor effects, consistent with our hypothesis, the APIM models revealed seven significant actor effects wherein higher level of smartphone proficiency, smartphone duration, smartphone usage, and overall smartphone score associated with their own lower levels of loneliness for older couples, whereas wives’ smartphone usage wasn’t significantly associated with their own loneliness. The key factors related to one’s own loneliness were smartphone duration for husbands and smartphone proficiency for wives, respectively. In general, our finding indicated that smartphone use was related to lower loneliness in older people, which is consistent with previous studies. For example, Petersen et al. found in a cohort study that increased loneliness among older adults in America was associated with decreased daily phone use, and was more closely linked to incoming than outgoing phone calls [5]. In the research examining the association between use and gratifications of mobile phone and loneliness, Wang et al. reported that mobile phone usage may influence social connections by enhancing or undermining the face-to-face social interaction, thereby having a positive or negative effect on loneliness [7]. It is worth mentioning that in the context of the COVID-19 pandemic, public health control measures such as masking, physical distancing, and limited contacts might be associated with reduced social interactions and therefore increased social isolation and loneliness of the older adults [37]. A study of French people over 80 years old found that smart devices such as smartphones and computers could help the older adults maintain a social links and interact with relatives during the COVID-19 pandemic, even though most older people preferred face-to-face interact, with important implications for reducing social isolation and loneliness [38].

As for partner effects, only two significant partner effects were found to support our hypothesis, one from husbands’ smartphone duration to wives’ loneliness, and the other from husbands’ overall smartphone score to wives’ loneliness. Specifically, the more time husbands spent on smartphones per day, or the better they used their smartphones overall, their wives would respond with less loneliness. One possible mechanism underlying this partner effect is emotional contagion, whereby a husband's positive emotional state from smartphone use may spread to his wife, thereby reducing her loneliness. There are several possible reasons why husbands’ smartphone behavior may be related to less loneliness of their wives. It is possible that due to the inherent interdependence of the marital relationship, loneliness may spread to and from spouse [39,40], in the same way, husbands’ satisfaction and happiness from smartphone use might spread to their wives, hence, the wives’ loneliness was also somewhat reduced. As to the reason why this effect was not bidirectional, we speculate that husbands seem to have greater impact on wives than wives do on husbands, especially for negative emotions such as loneliness, as mentioned in Ferrer and Nesselroads [41]. In addition, husbands have been proposed to be particularly sensitive to stress in marriage because of their lower tolerance for prolonged negative emotional states [42]. Due to the absence of prior study on the partner effect of smartphone behavior on loneliness, it is difficult to compare the results with similar studies, and future studies are required to confirm this effect.

It should also be noted that the duration model showed suboptimal fit (CFI = 0.829, RMSEA = 0.086), which may affect the interpretability of the partner effect from husbands’ smartphone duration. Caution is warranted when drawing conclusions from this specific model.

In the context of digital aging, it is important to understand the impact of smartphone usage behaviors on geropsychology within the framework of social relationship networks. Different cultural contexts may reshape the mechanisms of action. From a theoretical standpoint, this integration of family systems theory and digital behavior research provides new ideas for digital health interventions in aging societies worldwide. Because there is a lack of previous research on how smartphone usage affects loneliness, it is difficult to compare the results with those of similar studies. Subsequent research could compare the boundary conditions of the “companion effect” across cultures or explore how technology design can adapt to older couples’ interdependence needs.

## Limitations

There are several limitations that should be mentioned when interpreting the findings. First, data from this cross-sectional study limit causal inferences; we can only report associations. Second, this study did not examine the possible inverse relationship between smartphone behavior and loneliness. Third, communication and relationship quality were not measured, but could be important moderators of loneliness. Fourth, since this study only investigated 202 urban older couples in Chengdu, China, the findings should be replicated and extended to improve generalizability or to understand regional and population differences. Finally, we did not collect response rate data, which is a limitation. Furthermore, the composite smartphone behavior score was calculated by simple summation of ordinal (proficiency, duration) and count (usage type) scales. While this additive approach provides an easily interpretable global indicator of smartphone use, it assumes equal weighting across the three dimensions and has not been formally validated against alternative weighting schemes (e.g., factor-analytic or theoretically informed weights). Future studies could explore latent variable modeling or weighted composite indices to more precisely capture the multidimensional structure of smartphone behavior among older adults.

## Conclusions

In conclusion, the present study provides a meaningful exploration of the actor and partner effects of smartphone behavior on loneliness in older Chinese couples, and the findings partially support our hypothesis that greater smartphone use by husbands and wives is associated with lower levels of loneliness for themselves and their spouses.

## Abbreviations

APIM: Actor-partner interdependence model.
